# The effects of COVID‐19 pandemic on early childhood care systems in Hawaii in 2020

**DOI:** 10.1002/puh2.48

**Published:** 2023-01-13

**Authors:** Jeffrey K. Okamoto, Keiko Nitta, Kirra Borrello, Shelbi Nakano

**Affiliations:** ^1^ Department of Pediatrics John A. Burns School of Medicine University of Hawaii at Mānoa Honolulu Hawaii USA; ^2^ Hawaiʻi Department of Health Honolulu Hawaii USA; ^3^ Department of Biology School of Life Sciences University of Hawaii at Mānoa Honolulu Hawaii USA; ^4^ Department of Public Health Studies Thompson School of Social Work & Public Health University of Hawaii at Mānoa Honolulu Hawaii USA

**Keywords:** child development, child health, COVID‐19, early childhood, Hawaii, lead screening, pandemic

## Abstract

The COVID‐19 pandemic caused many effects on the referrals to and the work of governmental agencies working with young children. This article describes the impact on the use of early childhood evaluations and services in the State of Hawaii. We looked at several nonpublic data sets from the Hawaii Department of Health and Department of Human Services, comparing the rates of early intervention referrals, lead level screening, childhood immunizations, and child welfare referrals in 2019 and 2020. We also describe effects on the work processes in various early childhood programs from the COVID‐19 stay‐at‐home and work mandates. There was a decrease in rates of referrals to early intervention and child welfare services. There was less lead level screening being done, and childhood immunization rates dropped. Some of these issues stem from clinicians not seeing children whose families were worried about bringing their children for routine appointments. These clinicians do a lot of the developmental and lead screening, and much of the immunizations for children. Home visiting programs were interrupted as they could not do in‐person screenings of families. Therefore, they could not easily identify families that benefit from the support of a trained home visitor. The Special Supplemental Nutrition Program for Women, Infants, and Children (WIC) could not do anemia checks or measurements on children's growth. With ongoing COVID‐19 issues in the State of Hawaii, remedies are required given ongoing effects on clinician offices practices and childhood program processes.

## INTRODUCTION

The COVID‐19 pandemic has caused deaths, affected hospitals and their services, and decreased tourism in Hawaii. The pandemic may not have caused the same level of severe illness or death in children as older adults, but there have been effects on children and families. For example, there have been many issues around the closing and reopening of schools, and the effects on children are more extensive than just the impact of school issues. This article examines a number of consequences on child development, particularly to the youngest and most vulnerable children in Hawaii during the COVID‐19 pandemic.

During the first year of the pandemic (2020), several systems for child welfare were affected. This included lead screening, developmental supports to children with delays/disabilities, provision of childhood immunizations, home visits to high‐risk families, and referrals for child abuse and neglect cases. There are downstream effects on the development of young children if support from such systems is interrupted. For example, if children are not identified to have been poisoned with lead, these children will suffer from the effects on their development from ongoing toxicity. If children are not given therapy for developmental delays, then these delays may persist or worsen and cause behavioral problems.

The COVID‐19 pandemic required a cessation of face‐to‐face services to decrease the risk of transmission of the virus. Most services transitioned to a virtual platform, which decreased, for example, the use of blood testing for anemia or lead. Developmental assessments and measurements of children could not be done in‐person. Families could not travel, due to prohibitions and quarantine requirements, and did not want to leave their homes due to the fear of virus infection. People on the neighboring islands outside of Oʻahu stayed on their home islands and therefore could not access services often delivered on Oʻahu (where the main city of Honolulu is). In this article, we analyzed Hawaii Department of Health (DOH) and Department of Human Services (DHS) data sets to look at the effects of the COVID‐19 pandemic on early childhood system processes. We describe the effects of the COVID‐19 pandemic on particular agencies that support young children and their families in Hawaii.

## EARLY INTERVENTION

The early intervention (EI) system in Hawaii and other US states provides services for children from birth to 3 years of age. The children being referred for services in the EI system have delays or disabilities that can be supported by occupational therapists, physical therapists, and speech‐language pathologists in addition to early education and other specialists. The positive effects of EI on motor abilities, child intelligence, and academic achievement have been shown over many years of research [1, 2, 3, 4, 5].

**FIGURE 1:**
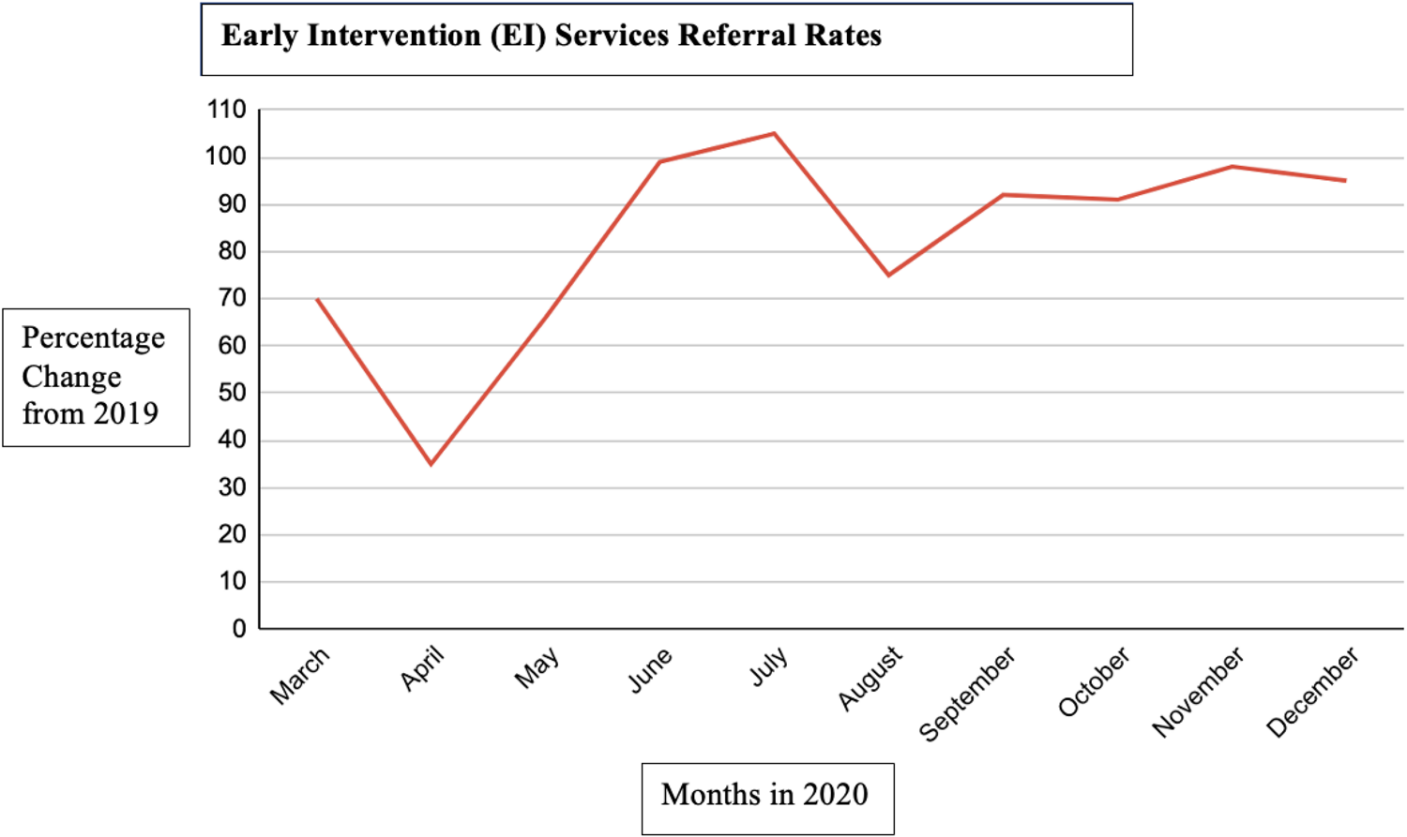
Effects of the COVID‐19 pandemic on early intervention (EI) services referral rates in the State of Hawaii, 2019–2020

EI services are mandated through federal law, which is Part C of the Individuals with Disabilities Education Act (IDEA). The IDEA law governs how state agencies provide EI, special education, and related services to children birth through 21 years of age with disabilities. Part C enrollment varies from state to state in this country [6]. Every child referred receives a multidisciplinary evaluation to determine eligibility for services. If eligible, the child and family receive support and services identified on their Individualized Family Support Plan. Every family is assigned a Care Coordinator who helps the family to navigate the EI system from referral through transition, including connecting them to other community resources. The DOH keeps statistics regarding EI referrals and enrollment. Enrollment has decreased from pre‐COVID levels in Hawaii (see Figure 1).

EI programs nationally are mandated by federal law to provide their services in “natural environments,” usually the parent/caregiver home. This is especially true in Hawaii where a parent/caregiver coaching model is used for services. The COVID‐19 pandemic has affected the face‐to‐face work with families in natural environments.

Hawaii's EI programs have been using the term “telepractice,” which in other communities is called “telehealth.” A paper from 2012 relates states being interested in participation in telehealth (telehealth is the term used in that paper) for EI because of personnel shortages and the need to serve in‐person hard to reach rural areas. At that time, 20% of the states were participating in telehealth activities for EI services [7]. A more recent report out of Colorado discussed more flexible scheduling for both provider and families [8]. There were also findings that telehealth supported a family coaching model, which is the model Hawaii uses. However, some service coordinators and providers related telehealth as being less effective, less personal, and less family friendly. In fact, a study conducted in Illinois in 2020 found that only 28% of EI providers reported high confidence in telehealth, which is seen through the significant decrease in the number of sessions delivered and the number of children per caseload during the COVID‐19 pandemic [9]. Nonetheless, this report relates some emerging research relating efficacy similar to in‐person and positive ratings by families.

## LEAD LEVEL SCREENING IN CHILDREN

The catastrophe in Flint, Michigan highlights the importance of exposure to lead and effects on early childhood development [10]. Elevated lead is known to affect intellectual quotient (IQ), academics, and behavior [11]. Screening for lead in Hawaii is dependent on primary care physicians (PCPs) who screen for lead exposure with questionnaires and, if necessary, blood tests. However, the Hawaii DOH's guidelines for lead screening are voluntary and currently recommend children be screened using a lead risk questionnaire and/or blood lead test at 9 months to 1 year and 2 years of age, and between age of 3 and 6 years, if risk for exposure increases or the child has never been tested before. A bill was introduced in the 2020 legislation mandating lead screening, but this has not yet been made law at the time of writing this article [12].

The COVID‐19 pandemic affected families who did not want to be seen in‐person for office visits with their PCP because of exposure to possible COVID‐19‐positive patients or staff. Also, many practices were shut down or decreased their visit numbers because of the pandemic.

**FIGURE 2:**
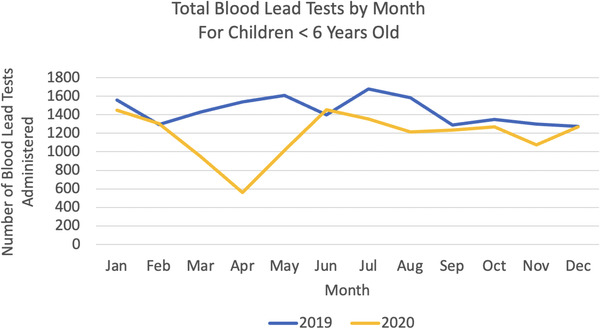
Effects of the COVID‐19 pandemic on lead level screenings among children under age 6 in the State of Hawaii, 2019–2020

Blood lead testing declined dramatically during the Spring shutdown with a 64% drop in April 2020. Testing numbers recovered in June 2020, and then dropped about 23% lower during the August surge in 2020. Testing declined a total of 18% (3160 tests) in 2020, and these yearly numbers provide the best clue as to the extent of the problem (see Figure 2).

## CHILDHOOD IMMUNIZATIONS

Childhood immunizations prevent a wide variety of conditions, including severe infections that can cause meningitis, hearing loss, and pneumonia. Preventing these infections therefore preserves children's brain functioning and/or hearing in those that would have been infected. Vaccine‐preventable diseases are costly, resulting in doctor's visits, hospitalizations, and premature deaths. Sick children can also cause parents to lose time from work.

Because of the effects of COVID‐19 on PCP operations and visits available for children, there were major decreases nationally and in the State of Hawaii in children getting their immunizations. In April 2020, national rates of the immunizations that were being purchased were down by 47% and Hawaii's immunizations that were being purchased were similarly down by 46% [13]. The flu vaccine in particular is an issue, as the flu may mimic COVID‐19 infection and so decreased immunization rates may increase costs of testing for possible COVID‐19 and unnecessary quarantine because of the flu. The Hawaii DOH, which advises the Department of Education (DOE) on immunizations required prior to school entry, did not change the requirements on immunizations for school entry.

Since many months had passed since the beginning of the pandemic prior to the start of the 2020–2021 school year, PCP clinics opened up, albeit with social distancing and barrier hygiene methods now put into place. Some pediatric clinics targeted children who were going into kindergarten and seventh grade for prioritized visits to help satisfy the DOE requirements for immunizations. A recent article from New York relates some rebound in immunizations after the initial drop in immunization rates [14].

## HOME VISITING

Families enrolled in home visiting are supported by periodic visits from a trained home visiting professional who encourages practices and interventions focused on child and family wellness, including parental education, health promotion, and linkage to needed community resources. The Hawaii DOH administers the legislatively established Hawaii Home Visiting Network, branded the “Your ʻOhana” Network, which is a public–private partnership with home visiting programs that strengthens families and promotes positive parent‐child relationships. Services are available prenatally and generally end at age 3 or at kindergarten entry. The goals of home visiting services are to (1) improve maternal and child health, (2) prevent child abuse and neglect, (3) promote child development and school readiness, and (4) encourage positive parenting [15]. Such programs show positive effects on interactions between parents and children, child development, and decreasing partner violence [16].

The social distancing requirements forced the home visiting programs to pivot from in‐person to virtual visits, which required adaptations to all aspects of service delivery including observations and screening. Home visiting programs were authorized to spend their contract funds to provide devices and internet connections for families in order to continue receiving home visits during the pandemic.

Prior to the COVID‐19 pandemic, the family resource specialists (FRSs) went to local birthing hospitals and local community events to screen and find new mothers to determine eligibility for home visiting services. Due to social distancing measures, FRSs also had to pivot screening virtually. Birthing hospitals provided lists of new mothers along with their contact information. Therefore, FRSs could not conduct their screenings in‐person in the hospital. This is secondary to hospitals limiting the number of nonclinical visitors and outside agency staff to decrease the COVID‐19 infection risks in their birthing units.

## WOMEN, INFANTS, AND CHILDREN PROGRAM

The Special Supplemental Nutrition Program for Women, Infants, and Children (WIC) is a program for states to provide supplemental food, health care referrals, and nutrition education for low‐income pregnant, breastfeeding and nonbreastfeeding women after delivery and to infants, toddlers, and children up to age 5 years who are found to be at risk for nutritional issues [17]. There is evidence showing cognitive benefits for children whose mothers participate in the WIC program [18].

The WIC program is usually a direct service program and sees families in‐person, providing nutrition education and taking heights and weights in their clinics. All services were face‐to‐face pre‐COVID‐19 period. Children were also given blood testing for anemia, a condition that can affect the learning of a young child. When the COVID‐19 pandemic hit, WIC program offices were shut down across the country and turned to remote services, providing all WIC program services via email, phone, and/or video appointments, interactive texting with WIC staff, and online education. Hawaii WIC program offices went virtual, which then decreased the number of child body measurements and anemia checks to zero.

Nationally, WIC programs had already started the implementation of a $8.5 million WIC Telehealth Innovations Project launched in 2019 (Public Law. 116‐6) [19]. Therefore, like EI, WIC in Hawaii is required to implement improved telehealth services.

A study done in California reported women having high levels of satisfaction with WIC's remote service delivery. In fact, 84% of participants rated the quality of WIC program services as being the same or better than before the COVID‐19 pandemic [20]. Researchers also found that delivering services remotely addressed existing barriers to WIC participation, such as lack of transportation, potentially long wait times, and the need to take off from work. Although the COVID‐19 pandemic forced WIC offices across the nation to pivot and serve families virtually instead of in‐person, this may have introduced a new way of delivering services due to the overall positive feedback from WIC participants.

Given the issues around food in Hawaii, WIC services are even more essential [21, 22]. A community poll showed that more than one in five Hawaii residents reported that “the food they bought just didn't last and they did not have money to buy more.”

## CHILD WELFARE REFERRALS

The Child Welfare Services (CWS) is part of the DHS Social Services Division. CWS provides support to children and their families when children are reported or are at risk of abuse and/or neglect. These services include child protection, preventive services, and diversion services to keep children out of foster care. CWS also has responsibilities over family support, foster care, adoption, independent living, adoption and guardianship payments, and licensing of resource caregivers (foster parents), group homes, and organizations placing children into foster and adoptive settings.

CWS's mission is to ensure the safety and wellbeing of children in their own homes first or, when necessary, in out‐of‐home placements. In any case where a child cannot be safely returned to their family within a certain time frame, CWS will establish a permanent placement for the child by means of adoption, legal guardianship, or long‐term substitute care. The local CWS offices are located on the islands of Oʻahu, Hawaii, Kauai, Maui, Molokaʻi, and Lanai [23].

All CWS offices remained open throughout the pandemic with protocols in place involving mask wearing, screening questions, and social distancing along with other cleaning protocols. Access to personal protective equipment was challenging initially. However, through collaborations with different agency partners along with donations and staff efforts involving making masks, CWS secured face masks. CWS received Coronavirus Aid, Relief, and Economic Security Act funds through the federal government to assist with the purchasing of masks, gloves, air filters, and prepaid cell phones along with data cards to help support virtual visits between parents and children.

Initially, there were practice changes implemented due to the uncertainty of the pandemic. Between March and June 2020, ongoing monthly visits to children and families involved with CWS were done virtually along with parent–child visits, known as ʻOhana Time', which was also conducted virtually between March and August 2020. The assessments of reports of abuse and neglect were continued face to face with the addition of screening questions, wearing of personal protective equipment during visits, social distancing, and meeting outdoors versus indoors when available.

A study that involved interviewing US‐based CWS staff showed participants feeling that the COVID‐19 pandemic compounded their baseline stress and verbalized their concern about how this will negatively impact their clients. In fact, studies have shown that these stressors among CWS staff, including high levels of burnout, compassion fatigue, and vicarious trauma, will likely have negative repercussions with regard to child outcomes [24]. CWS is a critical safety net for children who are in neglectful and/or abusive situations or part of child or labor trafficking. Referrals are particularly from schools, which are needed to provide virtual learning for the 2020 pandemic year. The decrease in referrals because of the pandemic are evident as shown in Table 1.

**TABLE 1:** Child welfare referrals by Department of Education (DOE) teachers in the State of Hawaii, 2019–2020

CWS referrals			
Months	Teacher referrals in 2019	Teacher referrals in 2020	Total referrals in 2020 (teacher and nonschool referrals)
January	35	40	274
February	31	45	263
March	29	27	260
April	41	0	179 (down 33%)
May	34	4	238 (down 11%)
June	4	2	203 (up 35%)

*Source*: Hawaiʻi Department of Human Services.

Young children have their parents, caregivers, and other family members and a number of supports that ensure their safety, growth, and development. Because of the economic stresses from the COVID‐19 pandemic, many families lost their sources of income or occupation. Families that may have already been under stress pre‐COVID‐19, such as those with single parents, had their stresses exacerbated by their children mandated to be home from school.

## CONCLUSIONS

The COVID‐19 pandemic posed many challenges to the child care systems in Hawaii. There was a drop in referrals for EI services for children with developmental delays or disabilities. There was a decrease in lead level screening and immunization in children. These all were probably affected by clinicians not seeing the numbers of children secondary to clinics decreasing their well‐child care visits, or families not wanting to see their clinicians for routine visits. These visits usually include developmental screening, lead screening, and immunization administration. Home visiting programs were not allowed into hospital birthing units to screen for mothers who were of higher risk to domestic violence, drug use, etc. Therefore, they could not easily identify families for their programs. Home visiting programs also went from in‐person to virtual visits, which changed how they did observations and screening. WIC programs went virtual, which made it difficult if not impossible to measure children's growth and do blood checks for anemia. Child welfare systems were affected, not being able to meet in‐person with families reported to have abused or neglected their children. Additionally, parent–child visitations went virtual. As a response, state agencies, nonprofit organizations, and clinics have adapted and adjusted to the new realities while the pandemic continues. There are concerted efforts to improve child health care in Hawaii by strengthening the referral and support systems of care around young children.

## AUTHOR CONTRIBUTIONS

Jeffrey K. Okamoto: *Conceptualization, writing, original draft, and review and editing*; Keiko Nitta: *Conceptualization and data curation*; Kirra Borrello: *Methodology, writing, and original draft*; Shelbi Nakano: *Writing, review, and editing*.

## CONFLICTS OF INTEREST

The authors declare no conflict of interest.

## HUMAN PARTICIPANT PROTECTION

This study was not human participant research.

## ETHICS STATEMENT

This material is the authors' own original work, which has not been previously published elsewhere. The paper is not currently being considered for publication elsewhere. The paper reflects the authors' own research and analysis in a truthful and complete manner.

## Data Availability

Data are available on request from the authors.
